# Men in Mental Health: A Scoping Review of Challenges, Contributions, and Future Possibilities of Recruiting into Nursing

**DOI:** 10.3390/nursrep15030097

**Published:** 2025-03-13

**Authors:** Natasha Reedy, Trish Luyke, Rowena McGregor, Rachel King, Rhonda Dawson, Brendon Robinson, Daniel Terry

**Affiliations:** 1 School of Nursing and Midwifery, University of Southern Queensland, Toowoomba, QLD 4350, Australia; trish.luyke@unisq.edu.au (T.L.); rhonda.dawson@unisq.edu.au (R.D.); brendon.robinson@unisq.edu.au (B.R.); daniel.terry@unisq.edu.au (D.T.); 2 Centre for Health Research, University of Southern Queensland, Toowoomba, QLD 4305, Australia; 3 Support for Learning, University of Southern Queensland, Toowoomba, QLD 4350, Australia; rowena.mcgregor@unisq.edu.au; 4 School of Mathematics, Physics and Computing, University of Southern Queensland, Toowoomba, QLD 4350, Australia; rachel.king@unisq.edu.au; 5 Darling Downs Hospital and Health Service, Toowoomba, QLD 4350, Australia; 6 Institute of Health and Wellbeing, Federation University, Ballarat, VIC 3350, Australia

**Keywords:** career choice, male nurses, men in nursing, mental health nursing, nurse retention, workplace violence

## Abstract

**Background/Objectives:** Historically, male nurses were predominant in mental health settings due to their perceived physical strength and ability to manage violent patients. However, societal changes and the evolution of nursing education have led to a decline in male participation. This study aims to explore the characteristics, qualities, and attributes of male mental health nurses, while aiming to identify factors that attract and retain, as well as that deter, men in this field, to inform male recruitment and retention strategies to grow the mental health nursing workforce. **Methods:** A scoping review was conducted across six databases, including PubMed, MEDLINE, Web of Science, Scopus, CINAHL, PsycINFO, and ProQuest. The focus was on studies from 1970 to 2024. Screening and selection of studies were based on eligibility criteria. Narrative synthesis was conducted, and the study follows the PRISMA for Scoping Reviews checklist. **Results:** Limited research exists on male mental health nurses. The data highlight the unique contributions of male nurses, including their resilience, teamwork, and emotional competence. They also identify challenges such as workplace violence, stigma, and lack of career development opportunities. The literature suggests that targeted recruitment strategies and supportive work environments are essential to increase the number of male mental health nurses and address the nursing shortage in this specialty. Positive academic experiences and professional development opportunities are crucial for retaining male nurses. **Conclusions:** Addressing stigma associated with mental health nursing is needed, starting with a positive public health education campaign. Addressing workplace violence needs to stem from improved organisational procedures that promote the safety and wellbeing of nurses and clients, combined with de-escalation education and training; mentoring are vital to improving attraction, job satisfaction, and the retention of male nurses. By understanding these factors, health care organisations can better support male mental health nurses and enhance the overall quality of mental health care.

## 1. Introduction

Mental health is integral to an individual’s wellbeing and consequently is a valued asset for individuals and families, workplaces, and the community. The World Health Organization [1] (p. 1) defines mental health as a “state of mental well-being that enables people to cope with the stresses of life, realize their abilities, learn well and work well, and contribute to their community”. Currently, mental illness is a major contributor to the global disease burden, affecting 1 in 6 years lived with a disability impacting people in all facets of their life, inclusive of comorbidity and mortality [1]. Mental health nursing is a specialty area of nursing and provides care from a holistic and person-centred care approach [2]. Despite the prevalence and consequences of mental illness, very few individuals are attracted to mental health nursing as a career due to the associated stigma from the broader community and within the nursing profession [2,3]. Thirty years on, there is yet to be a tangible solution globally and remains a significant gap in the literature. If the nursing workforce supply is left unaddressed, further harm to people and the broader community will occur.

Historically, mental health has been a separate discipline from general nursing, with males predominantly forming the workforce and serving as a cornerstone of mental health care [4,5]. Understanding their legacy and the qualities and attributes they have brought to the profession are important for creating a strong identity for nurses who are male and in mental health. It is vital to recognise how these qualities are suited to this specialist field [4,5] to grow the mental health nursing work force and raise the level of awareness of the vital role mental health nurses have in client care. To address this concern, this research study begins by contextualising the historical contribution of male mental health nurses to identify the unique qualities and attributes they possess, what attracts and retains them, and why they might think about leaving the mental health setting.

### 1.1. Background

Historically, psychiatric mental health nursing was predominantly the domain of nurses who are male [4,5,6]. Before the development of neuroleptic medications in the 1950s, asylums were created to confine the seriously mentally ill who could not be accommodated in society, and males had an advantage in terms of strength [7]. In the US, during the mid-1700s, both male and female ‘lunatics’ were admitted to general hospitals and cared for by male attendants. Reports of rough handling are consistent with the view of mental illness at that time [8].

The passing of the Lunacy Act in 1845 in England led to the construction of asylums throughout the country, which then became the model for European countries [9,10]. Asylum staff were referred to as servants, keepers, or attendants. They had no formal training, were viewed as only slightly better than the patients, and came from the lowest social classes [6,9,10]. Often, attendants shared common living spaces with the patients, making the position unattractive and contributing to high turnover [10]. This practice continued in America until the 1920s [11].

During the 1870s and 1880s, asylums needed to reflect the changing view of mental illness and align with general hospitals [10]. Mental health nursing emerged under medical control, with middle-class men managing asylums as stewards. Female wards were staffed by women and typically managed by the steward’s wife [10]. Wage increases, uniforms, and rostered days off began to elevate the status of attendants. Male staff continued to be referred to as attendants, while females began to be called nurses [6].

Formal training for mental health nurses began in Europe during the 1880s and 1890s, modelled after the general nursing training of Florence Nightingale and utilising textbooks written by doctors [9,10]. Nightingale was one of the first to recognise that different training was essential for treating ‘lunatics’, also suggesting men as her ‘best observers’ for these patients [8], and observation was considered best practice when providing care.

Nurses who were male were unwelcomed in general nursing and viewed as inferior, and mostly worked in asylums [12]. Larger English asylums offered a Medico-Psychological Association (MPA) two-year training certificate, though training was not compulsory and was not recognised as nursing training by the General Nursing Council (GNC) in England. Despite this, Registered Mental Nurses were added to a supplementary section of the GNC registers later in 1919 [12]. Conversely, US mental health nursing training began in 1882 with the opening of the first school of nursing in a mental hospital, admitting male students. Nurses who were male were employed to deal with violent patients and protect female asylum workers. The Asylum Workers Association was formed in 1910 but did not develop an association with the American Psychiatric Association until the 1930s [11].

In 1923, the British Supplementary Register for Mental Nurses was established under the General Nursing Council [13]. By the mid-20th century, a hierarchy of mental nurses was established, with double-trained nurses (general and mental) being called psychiatric nurses, while others remained mental nurses or attendants. Then, during World War I, in England, in response to male staff shortages, nurses who were female began to work in male wards; however, nurses who were male were unable to care for female patients for many years to come [9]. The 1950s and 1960s in England heralded significant changes in mental health nursing organisation, with the inception of the ‘Society of Registered Male Nurses’, the ‘Society of Mental Nurses’, and the ‘Association of Chief Male Nurses’ [13].

Beyond the US and the UK, the Lunacy Act 1871 was passed in Australia and convicts, who had often taken these care roles, were replaced with both nurses who were male and female [14]. Formal training began in 1887 in New South Wales and Victoria, followed by Queensland in 1910 [14]. In 1928, mental health nurses who were male did not sit the educational standards test as their employment opportunities were limited [14]. Double certification for men was not encouraged, restricting nurses who are male to work in asylums and repatriation work. Training opportunities were not made readily available to mental health nurses who were male until the 1950s and without this training outdated practises and assaults on patients continued [14].

Between 1900 and 1950 in Australia, consistently more men became mental health nurses than women [14]. However, this phenomenon has since reversed, with the number of male mental health nurses and nursing graduates who are male declining [15]. The further decline in mental health nurse numbers has been attributed to the shift from direct-entry mental health nursing programmes to the inclusion of mental health nursing within general Bachelor of Nursing or Diploma of Nursing programmes, and is not just observed in Australia, but globally [16].

### 1.2. Problem Statement

The decline in mental health nurses has been identified and discussed since mental health nurse training was integrated into general nursing [17]. Thirty years on, there is yet to be a tangible solution globally and there remains a significant gap in the literature. Traditionally, mental health nurses who were male made up a significant portion of the mental health nursing workforce. Thus, identifying what attracts men to nursing and what prompts them to consider mental health nursing as a career pathway, along with the key factors that enable their retention in nursing, is essential to tap into the underutilised resource of males.

### 1.3. Aim

This scoping review aims to identify what are the characteristics, qualities, and attributes that make up mental health nurses who are male to inform targeted evidence-based recruitment strategies to increase the number of males undertaking mental health careers and help address the nursing shortage in mental health.

### 1.4. Objectives

In order, the addressed topics, aim of the review, and the key objectives include the following:

1. To identify the characteristics, qualities, and attributes of men working as nurses within the mental health setting.
2. To identify the factors that encourage and/or discourage the attraction and retention of men working as nurses within the mental health setting.

In addition, the research questions regarded the characteristics, qualities, and attributes of mental health nurses that are male in terms of attracting and retaining men in the mental health nursing setting.

## 2. Materials and Methods

The scoping review utilised narrative synthesis, as guided by Popay et al. [18], which was used to evaluate and synthesise textual findings from both quantitative and qualitative research to address the research aims and objectives. The analysis methods, inclusion criteria, and exclusion criteria were developed and documented, following the PRISMA for Scoping Reviews (PRISMA-ScR) checklist [19], to ensure the accurate and complete reporting of findings.

### 2.1. Search Strategy

To ensure the search strategy was comprehensive and well-documented, the Peer Review of Electronic Search Strategies (PRESS) checklist was used [20]. In addition, a health research-trained librarian (RM) provided suggestions and refined the search strategy, while also supporting the team with data searches. Overall, the process included verifying the translation of the research question into the search strategy, the correct use of Boolean and proximity operators, the inclusion of appropriate subject headings and text words, and checking for spelling, syntax, and line numbering errors. The selection of databases was reviewed to ensure they were appropriate for the research question, and any limits or filters applied were assessed to ensure they did not exclude relevant studies. By incorporating these elements, the search strategy is clear, comprehensive, and meets the requirements of the PRESS checklist [20].

Within this context, a broad literature search was conducted over July to October 2023, with a follow up review in August 2024 and January 2025 to address the aims of the study. Databases including PubMed, MEDLINE, Web of Science, Scopus, CINAHL, PsycINFO, and ProQuest were examined for publications between 1970 and 2024 to ensure the male nurse experience from the former hospital training era of the 1970s was captured. Articles, including grey literature, were further refined based on whether they had a full pdf and were in the English language. The databases were assessed using the title and abstract, followed by the full text. Key search terms and Boolean operators used for each database included the following: male, nurse, psychiatric, mental, insane, unit, hospital, asylum, institution, retention, attrition, and career, along with word variations and suffixes in line with the PRESS checklist (Appendix A). Hand searching and reference list checking were also undertaken to discover additional studies not initially captured.

### 2.2. Inclusion and Exclusion Criteria

**Figure 1:**
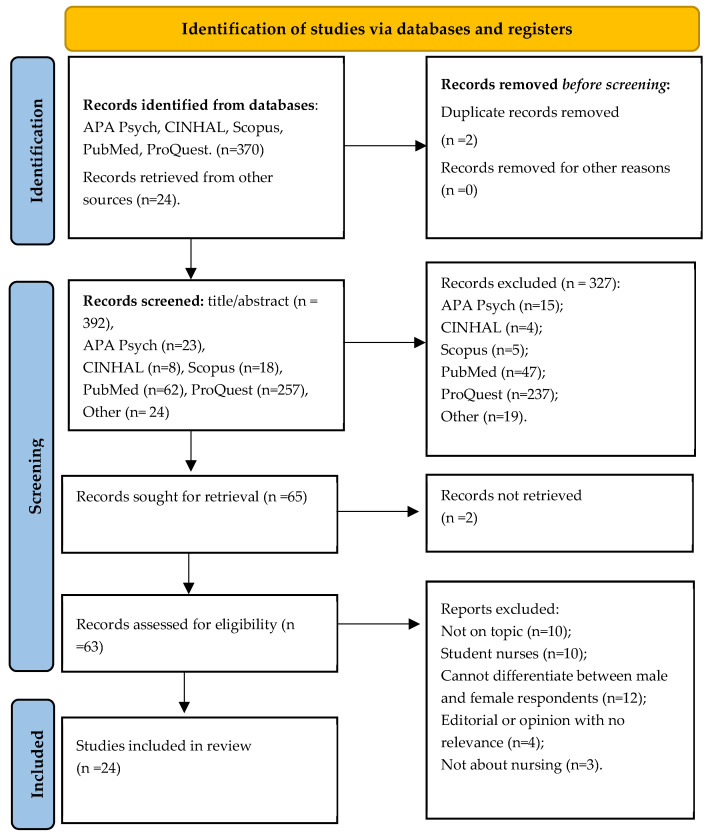
PRISMA flow diagram of search and study selection.

The reviewed studies included original research, international articles, and grey literature centred on mental health settings, and were inclusive of all registered nurses and enrolled nurses (diploma), and those who were male and employed in mental health careers. It must be noted that studies that included both male and females were included, but only where male data could be clearly identified or separated from female data. Studies were excluded if they were associated with student nurses, were articles associated with nurses who were female, were health professionals that were not nurses, or were assistants in nursing or similar. Further, studies were excluded if they were literature reviews and not original research. Lastly, full-text articles not published in English were not included (Figure 1).

### 2.3. Study Screening

All articles retrieved were exported and managed using EndNote (version 20) and were initially screened by one reviewer (TL) after duplicates were removed. All studies were initially screened based on titles, keywords, and abstracts to exclude irrelevant articles. In the second review round, the remaining full-text articles were then assessed independently by four reviewers (TL, NR, BR, RD) and judged against the inclusion and exclusion criteria. Each study was classified as ‘include’, ‘exclude’, or ‘not sure’ in the review. Any discrepancies between the two reviewers were resolved with a fifth reviewer (DT) until consensus was achieved.

### 2.4. Data Extraction and Analysis

Given the varied nature of the data, textual data extraction was conducted following the guidelines of Popay et al. [18]. Using a modified approach based on Colaizzi [21], each identified article was thoroughly reviewed multiple times to identify significant statements and meanings, while also developing interpretations, ideas, accounts, and assumptions based on the findings [18]. Common or recurring patterns and meanings among key statements were identified and aggregated during the review process. Additionally, textual data from each quantitative study were extracted due to the diversity in hypothesis testing, research questions, and findings, which made meta-analysis unfeasible. As data were extracted, findings were grouped into similar topics and domains, resulting in the identification of four overarching themes aligned with the study’s aims. Aggregation occurred when findings that conveyed the same understanding of the phenomena were grouped together to confirm the findings [18]. Conversely, configuration occurred when key findings that were thematically diverse and not suitable for data pooling were used to extend, explain, or counter-argue other findings, providing greater insights and understanding [18].

## 3. Results

The literature search yielded 394 potentially relevant publications and after removing duplicates (n = 2), including those that did not meet the inclusion criteria (n = 327), a total of 65 studies were agreed upon for inclusion in the literature review. Full texts were retrieved for all identified articles; however, two (n = 2) were not able to be retrieved. After full texts were reviewed, a further 12 were removed due to not being on topic, being related to student nurses, being editorial or option papers, and where there was difficulty differentiating between male and female data within the results (Figure 1). Overall, twenty-four papers were included: nine qualitative studies [15,22,23,24,25,26,27,28,29], seven cross-sectional studies [30,31,32,33,34,35,36], two ethnographic studies [37,38], two cohort studies [39,40], one scoping review [41], one longitudinal study [42], and one action research study [43]. The countries of origin were varied and included Australia, UK, USA, Israel, the Netherlands, and Taiwan. One notable characteristic was the age of the studies, with the majority of studies older than six years.

Overall, the review highlighted how there has been limited research on mental health nurses who are male. No metanalyses studies were located and there is a gap in the literature. From these studies, the following three themes were evenly presented, highlighting their relevance to the aim of this study (Table 1). Theme 1: Characteristics, qualities, and attributes of male mental health nurses. Theme 2: Why men are attracted to and stay in mental health nursing. Theme 3: Why men leave mental health nursing. Each of these themes are discussed in detail.

### 3.1. Characteristics, Qualities, and Attributes of Male Mental Health Nurses

Within the identified literature, the role of the mental health nurse was not clearly defined, and the professional identity of mental health nursing might be so weakly established that the future workforce lacks understanding of this specialist area [22]. Those already working in the field often define their practice in terms of values rather than skills [23,30]. Mental health nurses report seeing themselves as different from other nurses, and this difference is the highest level of satisfaction they achieve in their role. Alexander et al. [24] recognised that not everyone has the traits to be a good mental health nurse; some skills are learned, but others are inherent.

Further, Humble and Cross [26] (p. 133) identify that male mental health nurses have a “deep down desire to right the injustices”. They also describe male mental health nurses as being able to work with people rather than just perform tasks, share their feelings and thoughts with others, and be confident and competent when dealing with people with mental illness. Male mental health nurses are self-aware, curious, present themselves differently from general nurses, and are accepting of others.

In addition, McAllister et al. [33] asked current mental health nurses to identify inspirational attributes of mental health nurses and two domains were identified: firstly, an inspirational role model; secondly, passion, dedication, and commitment. One mental health nurse who was male was described as a “great leader… supportive, allowed us to practice to the limits of our knowledge and skills without interference.” [33] (p. 253). Another male mental health nurse was recognised for “instilling passion and enthusiasm through his style and charisma” [33] (p. 254).

Experienced mental health nurses express their desire for continued change to support consumers, including reducing stigma, feeling a sense of belonging and connection with colleagues, resilience, organisational support, and opportunities for professional development. These factors remain attractive aspects of their career choice. They also emphasise that a positive academic experience in mental health nursing contributes to longevity in the industry [28,33].

### 3.2. Why Men Are Attracted and Stay in Mental Health Nursing

The factors that attract nurses to mental health can be categorised into five main domains. Firstly, an interest in the field often develops due to content within mental health courses or positive exposure during clinical experiences prior to or while undertaking undergraduate degrees. This includes encouragement from facilitating mental health nurses who recognise potential in their students [24,30]. Secondly, many nurses are motivated by a desire to improve the circumstances of those challenged with mental illness, thereby making a meaningful difference in their lives [24,30]. Thirdly, personal or prior experience with individuals or family members who have mental illness can be a significant motivator, driving a deeper understanding and commitment to the field [24,26,30]. Fourthly, the opportunity to be holistically involved with patients on multiple levels—person-centred care, family-centred care, and sometimes community-centred care—is particularly attractive to male mental health nurses, allowing for a more profound impact on patient well-being [23,27]. Lastly, some mental health nurses find themselves specialising in this field by chance or due to a lack of other opportunities, leading to a fulfilling career as they discover a passion for mental health nursing [30].

When examining the key elements associated with retaining men in mental health nursing, there was a consensus within the literature that a positive team culture, camaraderie, and teamwork significantly enhanced retention [24,30,42]. Shared experiences and a collective focus on patient well-being, along with mutual support among team members, were central to the desire to remain in mental health nursing [24]. Additionally, staying in mental health nursing was linked to hopefulness about client success, involvement in facilitating practice changes, and workplace practicalities such as job satisfaction, greater opportunities, professional development, feeling valued, and achieving a good work–life balance [22,30,33]. These factors were often shared experiences not just among males, but also for females who were nurses in the mental health profession.

However, it was noted that men were less likely to experience burnout due to prolonged detached concern, emotional distance, better regulation of their reactions, and a greater capacity to objectively distance themselves from patients to provide support [23]. In some cases, detached concern was discussed in terms of men being suited to mental health nursing due to the skill set required for the role, where emotional competence was linked to commitment to the career pathway [31].

### 3.3. Why Men Leave Mental Health Nursing

The literature regarding why men were wanting to leave mental health nursing was limited; however, a factor that impacted the desire to leave was centred on workplace violence and males feeling obligated to step-in. It was highlighted that violence in health care is increasing and directly correlated with burnout, contributing to reduced enthusiasm for work, a desire to leave the nursing profession, or transfer to another position or area of nursing [45]. In addition to physical violence, mental health nurses who are male have been demonstrated to be more likely to experience verbal aggression and sexual harassment than their female counterparts who are mental health nurses [45].

When violence occurs, mental health nurses who are male often feel obligated to take on leadership roles during physical de-escalation situations, yet they frequently lack adequate support when these situations arise [28]. This expectation can place additional stress on nurses who are male, who may feel responsible for managing potentially dangerous situations without sufficient backup from colleagues and management. The lack of support can exacerbate feelings of isolation and vulnerability, making it difficult for nurses who are male to perform their duties effectively. Addressing these issues requires a concerted effort to provide better mentorship, support, and recognition for mental health nurses, ensuring they feel valued and equipped to handle the demands of their roles.

## 4. Discussion

The focus of the review was to identify the characteristics, qualities, and attributes that make up male mental health nurses to inform targeted recruitment strategies and address the nursing shortage in mental health. The role of mental health nurses is multifaceted and complex, often extending beyond traditional nursing boundaries [2]. The literature highlights several key aspects that contribute to the ambiguity and unique identity of mental health nursing. This ambiguity is partly because all nurses have a role in providing mental health care [2] but, specifically for nurses who have been educated and trained in the specialty of mental health, their involvement in interdisciplinary teams—which often include psychiatrists, psychologists, social workers, and occupational therapists—has contributed to the eroding and blurring of their role. While this collaboration can blur the lines of their specific role, the collaborative care approach enriches their practice by integrating diverse perspectives and expertise [46]. Another factor to consider is that the scope of mental health nursing is continually evolving due to the increasing recognition of the importance of mental health in overall health. This evolution can lead to role ambiguity but also offers opportunities for mental health nurses to expand their practice areas, such as in community settings, schools, and primary care [47]. This knowledge of role diversity is important to highlight when designing attraction and recruitment strategies, and when designing nursing programs’ theoretical and professional experience placements within the curriculum. Doing this ensures student nurses are adequately prepared and exposed to these areas to encourage thoughts of career specialisation.

All nurses are educated to deliver care using the nursing philosophies of holism and person-centred care [48,49,50]. However, mental health nurses emphasise the holistic approach to care, focusing on the emotional, psychological, and social aspects of health as a critical element in recovery care [2]. This approach aligns with their personal values of empathy, compassion, and patient-centred care [22]. This knowledge is important to leverage by organisations when designing attraction and retention strategies to promote job satisfaction and longevity in the workplace. Building strong therapeutic relationships is a core aspect of mental health nursing. These relationships are based on trust, respect, and mutual understanding, which are value-driven rather than purely skill-based [46].

Considering the broader negative societal stigma associated with mental health, males who enter this specialty demonstrate emotional resilience, which is a critical trait for mental health nurses, enabling them to cope with the emotional demands of their work. While some aspects of resilience can be developed through training and experience, an inherent capacity for empathy and emotional strength is often essential [24]. Additionally, mental health nurses must navigate diverse cultural contexts and understand the cultural dimensions of mental health. Cultural competence involves both intrinsic sensitivity to cultural differences and learned skills in culturally appropriate care [23]. Understanding this knowledge is vital in targeting a diverse population when attracting and recruiting males to ensure the clients feel culturally safe and to mitigate against vulnerability and fear of human rights violations [1].

Mental health nurses who are male often challenge traditional gender stereotypes in nursing, bringing unique perspectives and strengths to the field. Their presence can also encourage more men to consider careers in mental health nursing, contributing to a more diverse workforce, in addition to providing improved equity for clients who may prefer a male nurse and providing improved outcomes in their recovery [26,51]. Mental health nurses who are male can play significant roles in advocacy and leadership, addressing systemic issues in mental health care and promoting policies that support mental health initiatives [1,33].

Experienced mental health nurses who are male often serve as mentors and role models for new nurses or those who are exploring it as a career pathway, fostering a supportive learning environment [52]. Their passion, dedication, and commitment can inspire others to pursue and excel in mental health nursing [33]. Mental health nurses are increasingly involved in research and innovation, contributing to the development of evidence-based practises and new therapeutic approaches. Their insights and experiences are invaluable in shaping the future of mental health care [50]. Therefore, this knowledge highlights the importance of future research to partner with male mental health nurses to codesign attraction and recruitment strategies and the mental health curriculum to achieve robust outcomes.

Several factors attract men to mental health nursing and understanding these can help develop targeted recruitment strategies. Interest in the field often develops through exposure to mental health theoretical courses or positive clinical experiences during undergraduate studies [9,24,30]. This exposure may spark an interest for mental health nursing, as students gain firsthand experience and see the impact they may have in patients’ lives. Encouragement from experienced mental health nurses who recognise potential in students also plays a significant role. These mentors can provide guidance, support, and inspiration, helping students to see the value and rewards of a career in mental health nursing [30].

Many nurses are motivated by a desire to improve the lives of those with mental illness, driven by personal or prior experiences with mental illness in themselves or their families [23,24,30,53]. This personal connection can create a strong sense of purpose and commitment to the field. The opportunity to engage in holistic, person-centred care has been demonstrated to be particularly attractive to mental health nurses who are male, allowing them to make a profound impact on patient well-being [26,27]. This approach aligns with the values of empathy, compassion, and patient-centred care, which are central to mental health nursing and promoting positive client outcomes.

Some nurses find themselves specialising in mental health nursing by chance, discovering a passion for the field as they gain experience [30]. This serendipitous path can lead to a fulfilling career, as nurses realise the unique challenges and rewards of working in mental health. Understanding these factors is crucial for developing targeted recruitment strategies to attract more men into mental health nursing. By highlighting the opportunities for personal and professional growth, the importance of mentorship, and the impact of holistic care, recruitment efforts can be more effective in drawing men to this vital field and avoiding men discovering mental health nursing by chance and instead design a purposeful career promotion strategy.

In addition to these factors, societal attitudes towards gender roles in nursing can influence men’s decisions to enter the profession. Traditional gender stereotypes often portray nursing as a female-dominated profession, which can deter men from pursuing a career in nursing [53]. However, as more men enter the field and challenge these stereotypes, the perception of nursing as a gender-neutral profession is gradually changing [54]. This shift can encourage more men to consider mental health nursing as a viable and rewarding career option. However, more needs to be done from a government work planning level by creating a media campaign promoting the role of the nurse as gender diverse. Doing this may help attract males into the profession, create gender balance, and overcome the stereotyping that nurses are female.

Retention of mental health nurses who are male is influenced by a variety of positive factors, many of which are interconnected and contribute to a supportive and fulfilling work environment. These include a positive workplace culture, camaraderie, and teamwork. Additionally, shared experiences and mutual support among team members foster a sense of belonging and commitment to the field [24,30,42]. This sense of community is crucial, as it helps nurses feel valued and understood, reducing feelings of isolation and stress. Therefore, this knowledge is important to leverage by creating male support groups within nursing programmes and workplaces, especially for new males entering the mental health specialty.

Client success and involvement in practice changes are also important for retention. When nurses see positive outcomes in their clients, it reinforces their sense of purpose and satisfaction in their work. Additionally, being involved in practice changes allows nurses to feel that they are contributing to the improvement of their workplace and the care they provide, which can be highly motivating [22,30]. Job satisfaction, professional development opportunities, feeling valued, and achieving a good work–life balance are other critical factors. Opportunities for professional growth and development help nurses feel that they are advancing in their careers and acquiring new skills, which can enhance job satisfaction and retention [42]. Understanding this aspect is helpful when supervisors meet with the male nurses during annual performance reviews and support the employee to create a career pathway plan with key activities to enable evaluation and achievement.

Males working within mental health are often able to maintain emotional distance and regulate their reactions, which can reduce the likelihood of burnout. This ability to manage emotional stress is crucial in a field that can be emotionally demanding and helps sustain long-term commitment to the profession [31,39]. However, despite these positives, overcoming stereotypes and the stigma associated with mental health nursing remains a challenge. Nurses who are male may encounter societal and professional stereotypes that question their suitability for the role, which can impact their job satisfaction and retention [48]. Addressing workplace violence and stigma associated with mental health nursing is needed, starting with a positive public health education campaign.

Addressing these issues through mentorship programmes, continuous professional development, and positive academic experiences can enhance job satisfaction and retention. Mentorship programmes provide support and guidance, helping new nurses navigate the challenges of the profession and develop their skills. Continuous professional development ensures that nurses remain competent and confident in their roles, which can improve job satisfaction and reduce turnover. Positive academic experiences can also play a role in retention by preparing nurses for the realities of the profession and fostering a passion for mental health nursing [41].

Furthermore, organisational support and recognition are essential for retaining mental health nurses who are male. Organisations that recognise and reward the contributions of their staff can foster a positive work environment and enhance job satisfaction. Flexible work arrangements and policies that promote work–life balance can also help retain nurses by reducing stress and burnout [55]. By addressing these factors, health care organisations can improve the retention of male mental health nurses and ensure a stable and effective workforce. However, the onus is often left to the individual to manage and therefore is an area where improved organisational support needs to be implemented to ensure this is achieved through consultation, codesign, and evaluation.

Workplace violence is a significant factor impacting the desire of mental health nurses who are male to leave the profession. The increasing incidence of violence in health care settings is directly correlated with burnout, reduced enthusiasm for work, and a desire to leave the profession or transfer to another area [49]. Male mental health nurses are more likely to experience verbal aggression, sexual harassment, and physical violence than their female counterparts [45]. This heightened exposure to violence can lead to severe psychological consequences, including stress, anxiety, depression, and post-traumatic stress disorder [55].

When violence occurs, nurses who are male often feel obligated to take on leadership roles during physical de-escalation situations, yet they frequently lack adequate support [30]. This expectation places additional stress on nurses who are male, exacerbating feelings of isolation and vulnerability. The lack of support and recognition for their efforts can further diminish their job satisfaction and commitment to the profession. Addressing these issues requires a concerted effort to provide better mentorship, support, and recognition for all mental health nurses, particularly for those who are male and who may also be new to the mental health setting. Ensuring they feel valued and equipped to handle the demands of their roles is essential for retention.

Creating a supportive work environment involves implementing comprehensive workplace violence prevention programmes. These programmes should include training on de-escalation techniques, regular risk assessments, and clear protocols for reporting and responding to incidents of violence [55]. Additionally, fostering a positive team culture where nurses feel supported by their colleagues and supervisors can mitigate the negative effects of workplace violence. Peer support groups and counselling services can also provide emotional support and help nurses manage with the psychological impact of violence [3].

Future research needs to focus on the implementation of these attraction and retention strategies to leverage the positive aspects of being a mental health nurse as a male while simultaneously mitigating against the known negative aspects to attract males and those from diverse cultural backgrounds. It is also imperative to measure the effectiveness of these strategies annually using indicators such as male recruitment and retention numbers, job satisfaction, and career advancement.

### Limitations

This review focused on peer-reviewed empirical studies concerning male mental health nurses. While the aim was to include only articles discussing men in this role, some of the literature also included nurses who are female. In such cases, only data relevant to males were extracted. The review has additional limitations, such as only considering articles published in English and potential citation bias due to hand searching for relevant studies. The diversity and limited number of articles in terms of hypotheses, research questions, methodologies, study designs, and findings precluded a meta-analysis. These limitations may affect the generalisability of the review’s findings.

## 5. Conclusions

The scoping review highlights the significant challenges and contributions of male nurses in mental health settings. Historically, men played a crucial role in this field, but their numbers have declined due to societal changes and the evolution of nursing education. The review identifies key factors that attract men to mental health nursing, such as personal experiences with mental illness, the desire to make a meaningful difference, and positive academic and clinical experiences. Retention is influenced by a supportive team culture, professional development opportunities, and the ability to manage emotional stress effectively. However, the review also highlights the challenges faced by male mental health nurses, including workplace violence, stigma, and limited career development opportunities. Addressing these issues needs to begin with a positive public health education campaign, followed by targeted male recruitment strategies. While addressing workplace violence needs to start from improving organisational procedures that promote the safety and wellbeing of nurses and clients, combined with de-escalation education and training, promoting supportive workplace environments and robust mentorship programmes are essential to increase the number of men in mental health nursing and improve retention rates. By understanding and addressing the unique needs and contributions of male mental health nurses, health care organisations can better support this vital workforce and enhance the overall quality of mental health care.

## Figures and Tables

**Table 1:** An overview of the identified articles and themes from the findings.

Author (Year)	Country	Study Design	Study Aim	Sample Size	Findings	Themes
Alexander (2015) [24]	USA	Descriptive phenomenology	Exploring how nurses who are male in CAMHS experience differences in their gender performance	8 (4 men, 4 women)	Interest developed in nursing school, personal relevance, and validation of potential were key for career choice. Retention was influenced by overcoming stereotypes, positive team dynamics, and remaining hopeful.	1, 2
Crowther and Ragusa (2011) [15]	Australia	Focus group	Exploring the relationship between work stress, years of experience, and emotional competency	32 mental health nurses	Mental health nursing in rural Australia is in decline, affecting consumer outcomes and workplace experiences.	3
Fluttert et al. (2010) [39]	Netherlands	Cohort study (pre-test–post-test design)	The relationship between emotions and stress among mental health nurses	116 (men and women not specified)	Detached concern is meaningful in forensic nursing. Levels of concern did not change significantly after applying the early recognition method.	2, 3
Harrison et al. (2014) [30]	Australia	Cross-sectional design	To explore how mental health nursing could be promoted as a sustainable career option	192 mental health nurses (41.1%)	Staying in mental health nursing was affected by the following factors: facing reality, passion for mental health nursing, patient-centred caring, and workplace conditions.	1, 2, 3
Harrison et al. (2017) [22]	Australia	Qualitative study	Transition experiences of Indian nurses into the Australian mental health system	192 (men and women not specified)	Reducing stigma and improving visibility and recognition of mental health nursing can enhance recruitment and retention.	2, 3
Hazelton et al. (2011) [25]	Australia	Qualitative study	Factors influencing the transition and retention of mental health nurses during the initial years of practice	Mental health nurses (n = 18); clinical nurse consultants (n = 5)	New graduates face challenges and require support to navigate the complexities of mental health services.	3
Holyoake (2002) [37]	UK	Ethnographic study	Retaining the mental health nursing workforce: early indicators of retention and attrition	Not specified	Explores the construction and impact of male identity in nursing.	1, 2
Holyoake (2020) [38]	UK	Ethnographic study	Issues and challenges in mental health workforce development	Not specified	Nurses who are male navigate a balance between similarity and difference in gender performance compared to female colleagues.	1, 3
Humble and Cross (2010) [26]	Australia	Qualitative study	The lived experiences of veteran psychiatric nurses with more than 10 years’ experience in the psychiatric field to identify the factors that have influenced these nurses to continue to work in this area	Mental health nurses (n = 7)	Participants felt and saw themselves as different in many ways from other nurses. There was a high level of satisfaction achieved from their roles as they strived to achieve harmony.	1, 2
Humpel and Caputi (2001a) [31]	Australia	Cross-sectional	Associations between gender, sex, and caring behaviours among nurses in mental health	43 (men and women not specified)	Positive correlation between emotional competency and years of experience. Work stress impacts emotional stability.	1, 2
Humpel et al. (2001b) [32]	Australia	Cross-sectional	Incidence, type, related factors, and effect of workplace violence on mental health nurses	43 (men and women not specified)	Emotional competency and trait affectivity are related to stress and experienced emotions.	1, 2
Hung et al., 2014 [27]	Taiwan	Qualitative study	To understand the working experiences of novice psychiatric nurses during their first year in a clinical setting	Mental health nurses (n = 13 women, n = 2 men)	Nurses are often inadequately prepared for mental health nursing and have little understanding of mental illness, are unable to communicate appropriately with clients, and struggle to cope with the conditions.	3
Joseph, et al. (2022a) [41]	Australia	Scoping review	Exploring the relationship between work stress, years of experience, and emotional competency	n/a	Identifies factors affecting transition and retention, including support systems and workplace environment.	1, 2, 3
Joseph et al. (2022b) [28]	Australia	Qualitative study	Exploring how nurses who are male in CAMHS experience differences in their gender performance	Nurses trained in India (n = 16)	Indian nurses face cultural and professional challenges during transition into the Australian mental health system.	3
Lockhart (2004) [43]	Canada	Action research	Men in psychiatric nursing	6 (RNs = 3, students = 3)	Previous health work experience, knowing someone who works in psychiatric health, and commitment to and compassion for clients are qualities shared by the participants (all men).	1, 2
McAllister et al., 2010 [33]	Australia	Cross-sectional	To understanding the heritage of Queensland mental health nursing	Mental health nurses (n = 20)	Qualities included inspirational role model, and passion, dedication and/or commitment.	1
McCrae et al. (2014) [23]	UK	Qualitative study	To explore facilitators and barriers to professional identification in newly qualified nurses of accelerated post-graduate training	Mental health nurses (n = 9 women, n = 1 man)	Practise was defined in terms of values rather than skills and difficulty found in articulating a distinct role for mental health nursing. Experience in training and as a registered practitioner was considered rewarding; however, concerns were raised that nursing may not fulfil aspirations. Professional identity is likely to be a major factor in satisfaction and retention of nurses.	1, 2, 3
Robinson et al. (2005) [42]	UK	Longitudinal study	The relationship between emotions and stress among mental health nurses	Mental health nurses (n = 444, 66.8% women, 33.2% men)	Early indicators of retention include job satisfaction and support systems.	2, 3
Roche and Duffield (2007) [44]	USA	Expert opinion	Transition experiences of Indian nurses into the Australian mental health system	n/a	Workforce development is hindered by recruitment challenges and a lack of training opportunities.	1, 3
Shmilovitz et al. 2020 [34]	Israel	Cross-sectional	To study the association between sex types and caring behaviours in female and male nurses in the mental health field.	114 mental health nurses (n = 36 males, n = 78 females)	Androgynous female and male nurses dispalyed higher levels of caring than other sex types. Higher levels of caring were observed in female compared with male nurses overall and in behaviours of respect and attentiveness to patients’ needs and safety.	1
Thwala & Mokoena-de Beer, 2023 [29]	South Africa	Phenomenology	Psychological impact of violence on male nurses in forensic units in Gauteng, South Africa	11	Workplace violence causes physical and psychological injury. Fear of further injury impacts work attendance and ability to complete work tasks.	2, 3
Torkelson & Seed, 2011 [40]		Correlational design. Convenience sampling.	Gender differences in the roles and functions of inpatient psychiatric nurses	73 (28 male, 45 female)	Male and female nurses are differently impacted by perceived time spent on patient care, developing therapeutic and collegial relationships, and other nursing tasks.	1, 2
Yada et al., 2014, [35]	Japan	Survey: Nurse Job Stressor Scale and the Stress Reaction Scale of the Brief Job Stress Questionnaire	Differences in job stress experienced by female and male Japanese psychiatric nurses	244 (85 male, 159 female)	Male nurses impacted negatively by their self-perceived limitations regarding their psychiatric nursing abilities.	1, 3
Yang et al. (2018) [36]	Netherlands	Cross-sectional	Retaining the mental health nursing workforce: early indicators of retention and attrition	Mental health nurses (n = 245, 70% women, 30% men)	Workplace violence is prevalent and impacts mental health nurses’ well-being.	2, 3

## Data Availability

Data available upon request.

## Public Involvement Statement

No public involvement in any aspect of this research.

## Guidelines and Standards Statement

This manuscript was drafted against the PRISMA extension for Scoping Reviews (PRISMA-ScR) checklist.

## Appendix A

### Appendix A.1. APA Psych Info (EBSCOHOST) Search

Conducted on 31 August 2024
Date limit: 1970 upward
Other limits: peer reviewed, English language, full text

**Table A1:** APA pych info (ebscohost) search.

No	Query	Results
#1	male AND (nurs* OR nursing)	12,213
#2	#1 AND mental health	205
#3	#1 AND (mental health OR “psychiatric unit”)	1648
#4	#1 AND (mental health OR “psychiatric unit” OR mental asylum)	1906
#5	#4 AND career	1651
#6	#5 AND retention	1648
#7	male AND nurs* AND (mental health OR “psychiatric unit”) AND career AND retention OR attrition. Today’s number for this search = 30.	7443
#8	#5 AND (retention OR attrition) AND experience	2496
#9	#1 AND (“mental health” OR “psychiatric unit” OR mental asylum) AND career AND (retention OR attrition) AND experience	2481
#10	#1 AND (“mental health” OR “psychiatric unit” OR mental asylum) AND career AND retention AND experience	1633
#11	#1 AND (“mental health” OR “psychiatric unit” OR mental asylum)	1891
#13	“male nurs* AND (mental health nurs* OR psychiatric nurs*) AND (“mental health hospital” OR “psychiatric hospital”)	23

### Appendix A.2. CINAHL Search

CINAHL search conducted on 25 August 2024
Date limit: 1970 upward
Other limits: English language, full PDF

**Table A2:** Cinahl search.

No	Query	Results
#1	Male AND (nursing OR nurs*)	60,293
#2	#1 AND mental health	3687
#3	#2 AND career	96
#4	#3 AND retention	11
#5	#4 AND attrition	11
#6	#5 AND experience	10

### Appendix A.3. PubMed Search

PubMed search conducted on 25 August 2024
Date limit: 1970–2023
Other limits: full PDF, English language

**Table A3:** Pubmed search.

No	Query	Results
#1	Male AND (nurs* OR nursing) AND mental health	17,704
#2	Male AND (nurs* OR nursing) AND (mental health OR “psychiatric unit”)	19,670
#3	Male AND (nurs* OR nursing) AND (mental health OR “psychiatric unit” OR mental asylum)	52,085
#4	3 AND career	62
#5	4 AND retention	7
#6	3 AND (career OR retention)	7968
#7	6 AND experience	830

### Appendix A.4. SCOPUS Search

SCOPUS search conducted on 25 August 2024
Date limit: 1970 upward
Other limits: English language, full PDF

**Table A4:** Scopus search.

No	Query	Results
#1	male AND (nursing OR nurs*)	151,646
#2	#1 and mental health	13,865
#3	#2 AND career	239
#4	#3 AND retention	24
#5	#3 AND (retention OR attrition)	27
#6	#5 AND experience	18

### Appendix A.5. PROQUEST Search

PROQUEST search conducted on 23 January 2025
Date limit: 1970 upward
Other limits: Journals and theses/dissertations, English language

**Table A5:** ProQuest search.

No	Query	Results
#1	male nurse	703,902
#2	“male nurse”	5503
#3	male NEAR/5 nurse	26,609
#4	(male OR men OR man) NEAR/5 nurs*	187,685
#5	#4 TI OR AB	3345
#6	“psychiatric hospital”	59,013
#7	Psychiatric unit	453,878
#8	“Mental hospital”	30,674
#9	“Mental institution”	9433
#10	AND (hospital OR institution OR unit OR asylum)	2,702,157
#11	(psychiatric OR mental OR insane) in TI OR AB	576,242
#12	#10 TI OR AB	117,452
#13	#5 AND #12	1062
#14	abstract((male OR men OR man) NEAR/5 nurs* NOT student) OR title((male OR men OR man) NEAR/5 nurs* NOT student)	2784
#15	#13 AND #14	117
#16	#15 AND (career OR retention OR attrition)	35
#17	#11 AND #14	259
